# Dietary Supplementation with Cysteine during Pregnancy Rescues Maternal Chronic Kidney Disease-Induced Hypertension in Male Rat Offspring: The Impact of Hydrogen Sulfide and Microbiota-Derived Tryptophan Metabolites

**DOI:** 10.3390/antiox11030483

**Published:** 2022-02-28

**Authors:** Chien-Ning Hsu, Chih-Yao Hou, Guo-Ping Chang-Chien, Sufan Lin, You-Lin Tain

**Affiliations:** 1 Department of Pharmacy, Kaohsiung Chang Gung Memorial Hospital, Kaohsiung 833, Taiwan; cnhsu@cgmh.org.tw; 2 School of Pharmacy, Kaohsiung Medical University, Kaohsiung 807, Taiwan; 3 Department of Seafood Science, National Kaohsiung University of Science and Technology, Kaohsiung 811, Taiwan; chihyaohou@webmail.nkmu.edu.tw; 4 Center for Environmental Toxin and Emerging-Contaminant Research, Cheng Shiu University, Kaohsiung 833, Taiwan; guoping@csu.edu.tw (G.-P.C.-C.); linsufan2003@csu.edu.tw (S.L.); 5 Super Micro Mass Research and Technology Center, Cheng Shiu University, Kaohsiung 833, Taiwan; 6 Institute of Environmental Toxin and Emerging-Contaminant, Cheng Shiu University, Kaohsiung 833, Taiwan; 7 Department of Pediatrics, Kaohsiung Chang Gung Memorial Hospital and Chang Gung University College of Medicine, Kaohsiung 833, Taiwan; 8 Institute for Translational Research in Biomedicine, Kaohsiung Chang Gung Memorial Hospital and Chang Gung University College of Medicine, Kaohsiung 833, Taiwan

**Keywords:** chronic kidney disease, cysteine, hypertension, developmental origins of health and disease (DOHaD), renin–angiotensin system, gut microbiota, hydrogen sulfide, indole

## Abstract

Maternal chronic kidney disease (CKD) is linked to offspring hypertension. The gut microbiome and its tryptophan metabolites, nitric oxide (NO), and renin–angiotensin system (RAS) are closely related to the development of hypertension. Hydrogen sulfide (H_2_S) has shown an anti-hypertensive effect. Our objective was to test whether l- or d-cysteine supplementation in pregnancy can prevent hypertension programmed by maternal CKD in adult offspring and to explore the protective mechanisms. CKD was induced in pregnant Sprague Dawley rats by a 0.5% adenine diet for 3 weeks. l- or d-cysteine was supplemented at 8 mmol/kg body weight/day during pregnancy. Male offspring were sacrificed at the age of 12 weeks (*n* = 8 per group). Maternal CKD-induced hypertension was similarly prevented by l- or d-cysteine supplementation. The protective effects of l- and d-cysteine are related to reducing oxidative stress, rebalancing the RAS, and reshaping the gut microbiome. l-cysteine therapy protected adult offspring against hypertension and was associated with enhanced H_2_S production, restoration of NO bioavailability, enhancement of beneficial genera *Oscillibacter* and *Butyricicoccus*, depletion of indole-producing genera *Alistipes* and *Akkermansia*, and the reduction of several indole metabolites. d-cysteine treatment increased kynurenic acid, 3-hydroxykynurenine, and xanthurenic acid in the kynurenine pathway, decreased 5-hydroxytryptophan and serotonin in the serotonin pathway, and enriched genera *Bacteroides* and *Odoribacter* abundance. In summary, these results suggest that l- and d-cysteine protect against maternal CKD-induced offspring hypertension, likely by enhancing H_2_S production, modulating gut microbiota and its derived metabolites, and the restoration of NO and RAS.

## 1. Introduction

An increasing body of data highlights pregnancy and lactation as a critical period upon which maternal insults may shape health and disease in the resulting offspring, now referred to as the Developmental Origins of Health and Disease (DOHaD) [1]. Women with chronic kidney disease (CKD) are at risk not only for pregnancy-related but also offspring adverse outcomes [2]. Our prior research reported that maternal adenine-induced CKD induces blood pressure (BP) elevation in adult offspring, which coincided with alterations of gut microbiota composition, changes of derived metabolites, and increases of uremic toxins [3].

During pregnancy, the essential amino acid tryptophan is important for fetal development and placental protein synthesis [4]. Tryptophan metabolism undergoes three major pathways in the gut, leading to kynurenine, serotonin, and indole derivatives [5]. Indole formation occurs via the action of the enzyme tryptophanase [6]. Indole metabolites of tryptophan (i.e., indoxyl sulfate and indoleacetic acid) are a key group of gut microbiota-derived uremic toxins, which play a crucial role in the pathogenesis of CKD [6]. Tryptophan-derived uremic toxins can activate aryl hydrocarbon receptors (AHR) to induce oxidative stress through the activation of NADPH oxidase and the inhibition of antioxidant defense mechanisms [6,7]. It is well known that oxidative stress plays a key role in the pathogenesis of CKD and hypertension in developmental origins [8]. Considering the complexity of tryptophan metabolic pathways, the diverse properties of tryptophan-derived metabolites have been found to be associated with the pathophysiology of several diseases [5,6,9]. However, little information exists on whether tryptophan-derived metabolites are beneficial or harmful to maternal CKD-induced hypertension in adult offspring.

Recent research suggests that hydrogen sulfide (H_2_S) may have some health benefits as a reprogramming strategy, including an anti-hypertensive effect [10,11]. Several mechanisms have been reported underling its BP-lowering effects [12,13], including enhancing bioavailability of nitric oxide (NO), modulating the renin–angiotensin system (RAS), and reducing oxidative stress. We previously demonstrated that high salt-treated spontaneously hypertensive rats (SHRs) supplemented with l- or d-cysteine, precursors of H_2_S, between 4 and 6 weeks of age did not develop hypertension at 12 weeks old [14]. In addition to H_2_S generation, l-cysteine acts as a reduced glutathione (GSH) precursor; GSH is a well-known antioxidant [15]. Accordingly, l- or d-cysteine has antioxidant properties as a counterbalance to oxidative stress. In view of the fact that H_2_S has vasodilator properties and H_2_S can regulate microbial tryptophanase activity [10,16], we aimed to examine whether maternal l- or d-cysteine supplementation can afford protection for offspring rats against hypertension induced by maternal CKD and elucidate underlying mechanisms with a focus on gut microbiota and tryptophan-derived metabolites.

## 2. Materials and Methods

### 2.1. Animal Care and Experimental Design

Virgin Sprague Dawley (SD) rats were used at the beginning of study (8 weeks of age, purchased from BioLASCO Taiwan Co., Ltd., Taipei, Taiwan). On arrival, the rats were housed in our AAALAC full-accreditation animal facility. The procedures used in this study were conducted according to the rules of Care and Use of Laboratory Animals of the National Institutes of Health and the IACUC of Chang Gung Memorial Hospital (Permit # 2020073102).

To induce a CKD model, female SD rats received regular chow (*n* = 8) or chow supplemented with 0.5% adenine for 3 weeks in accordance with our previous work [3]. Female rats were caged with male rats until mating. After confirmation of mating by the presence of a copulatory plug, the dams were housed individually and randomly assigned into four groups: control, CKD (adenine-treated rats), LC (adenine-treated rats received l-cysteine supplemented at 8 mmol/kg body weight/day during pregnancy), and DC (adenine-treated rats received d-cysteine supplemented at 8 mmol/kg body weight/day during pregnancy). The doses of l-cysteine and d-cysteine used here are based on our previous study conducted in rats [14]. Litter size standardization was carried out and litters were culled to eight pups. Because males have been found to have hypertension at a younger age than females [17], only male progeny was selected from each litter for use in subsequent experiments. Male progeny was assigned to four experimental groups (*n* = 8 per group): C, CKD, LC, and DC. Pups were weaned at 3 weeks onto normal chow.

Rats were acclimated to the CODA non-invasive blood pressure system (a tail-cuff method, Kent Scientific Corporation, Torrington, CT, USA) for 20 cycles per rat for one week prior to the experiment, to ensure accuracy and reproducibility. According to our protocol [3], BP was measured in conscious rats every four weeks. A total of 32 offspring were sacrificed at 12 weeks of age. Fecal samples were collected in the morning prior to sacrifice by lifting the tail and twisting it towards the back to induce defecation. Later feces samples were stored at −80 °C in a freezer until extraction. Blood samples were collected in heparin tubes. The kidneys were harvested and stored at −80 °C until analysis. Kidneys were harvested after perfusion with phosphate buffered saline. One kidney was removed and divided into cortex and medulla and snap frozen; the other kidney was fixed and removed for immunohistochemistry. 

### 2.2. High Performance Liquid Chromatography–Mass Spectrometry (HPLC-MS/MS)

We used an Agilent Technologies 1290 high performance liquid chromatography (HPLC) system coupled with an Agilent 6470 Triple Quadrupole liquid chromatography tandem mass spectrometry (LC/MS, Wilmington, DE, USA) to determine plasma levels of H_2_S and thiosulfate as described previously [18]. The H_2_S derivative sulfide dibimane (SDB) and thiosulfate derivative pentafluorobenzyl (PFB)-S_2_O_3_H were determined. The detection of target compounds was conducted in the selected reaction monitoring mode using transitions of *m/z* 415→223, *m/z* 292.99→81, and *m/z* 212.99→93, for SDB, PFB-S_2_O_3_H, and PHB, respectively. We used phenyl 4-hydroxybenzoate (PHB) as an internal standard.

### 2.3. Liquid Chromatograph Tandem–Mass Spectrometry (LC-MS/MS) 

The plasma levels of tryptophan and its metabolites were analyzed by LC-MS/MS. A total of 13 tryptophan metabolites were determined, including kynurenic acid, xanthurenic acid, 3-hydroxykynurenine (3-HKN), 3-hydroxyanthranilic acid (3-HAA), 5-hydroxytryptophan (5-HTP), serotonin, hydroxyindole acetic acid (5-HIAA), N-acetylserotonin (N-AS), indoxyl sulfate (IS), indole-3-acetamide (IAM), indolelactic acid (ILA), indole-3-carboxaldehyde (ICA), and indoleacetic acid (IAA). Plasma samples (200 μL) were added into the 1.5 mL centrifuge tube containing 400 μL internal standard mix solution, 200 µL acetonitrile, and 400 µL methanol. Tubes were placed on a centrifuge for 15 min at 14,000 rpm in 4 °C. The supernatant was taken and concentrated to 100 μL by centrifugation. Later, 100 μL of 5 mM ammonium acetate aqueous solution and methanol (9:1, *v*/*v*) were added and mixed well. The sample was then injected into the LC-MS/MS at 2 μL. Separation was performed on chromatography using an Agilent 1200 Infinity II HPLC system equipped with a Water Acquity UPLC HSS T3 column (2.1 mm × 100 mm; 1.8 um; Agilent Technologies). The components were eluted by a gradient of solvent A (5 mM ammonium acetate aqueous solution) and solvent B (acetonitrile). The Agilent 1200 Infinity II HPLC system was coupled with an Agilent 6470A Triple Quadrupole LC/MS (Agilent Technologies). The eluate was monitored for tryptophan metabolites in multiple reaction monitoring (MRM) detection mode with characteristic precursors and product ions.

### 2.4. Quantitative RT-PCR

Rat kidney cortex tissue was homogenized in lysis buffer and total RNA was extracted using the TRIZOL method (Invitrogen, Carlsbad, CA, USA), as described earlier [3]. Two-step quantitative real-time PCR was performed using the QuantiTect SYBR Green PCR Kit (Qiagen, Valencia, CA, USA) on an iCycler iQ Real-Time PCR Detection System (Bio-Rad, Hercules, CA, USA) in duplicate. A total of four genes involved in H_2_S production were determined, including cystathionine-synthase (CBS), cystathionine-lyase (CSE), 3-mercaptopyruvate sulphurtransferase (3MST), and d-amino acid oxidase (DAO). We also measured several renin–angiotensin system (RAS) genes, including renin, (pro)renin receptor (PRR), angiotensin converting enzyme (ACE), angiotensin converting enzyme-2 (ACE2), angiotensin II type 1 receptor (AT1R), angiotensin II type 2 receptor (AT2R), and angiotensin-(1–7)/Mas receptor (MAS). We used the 18S ribosomal RNA (R18S) reference gene as the internal control. Each sample was run in duplicate. The primers were designed using GeneTool Software (BioTools, Edmonton, AB, Canada) and shown in Table 1. To determine relative gene expression, the comparative threshold cycle (Ct) method was used. The fold change for each mRNA relative to the control was calculated using the formula 2^−ΔΔCt^. 

### 2.5. Analysis of Gut-Microbiota Composition

As described previously [3], bacterial DNA from frozen stool specimens was extracted and analyzed by 16S rRNA metagenomics analysis at Biotools Co., Ltd. (Taipei, Taiwan) using an Illumina Miseq platform (Illumina, San Diego, CA, USA). The sequences were processed using QIIME version 1.9.1. Sequences with a distance-based similarity of 97% or greater were clustered into operational taxonomic units (OTUs) by USEARCH algorithm. The phylogenetic relationships were constructed based on a representative sequence alignment with FastTree. We compared patterns of α- and β-diversity for microbial communities. Alpha diversity was measured by ACE index. We assessed the β-diversity of the gut microbiota across groups using the Analysis of similarities (ANOSIM) and Partial Least Squares Discriminant Analysis (PLS-DA). The linear discriminant analysis effect size (LEfSe) was assessed to discover high-dimensional biomarkers. 

### 2.6. Analysis of Nitric Oxide Parameters

We used the HP Agilent 1100 HPLC System (Agilent Technologies Inc., Santa Clara, CA, USA) with fluorescence detection of O-phthalaldehyde/3-mercaptopropionic acid (OPA/3-MPA) derivatives to measure NO-related parameters in the plasma as previously described [3]. These parameters included l-Arginine and NO synthase inhibitor asymmetric and symmetric dimethylarginine (ADMA and SDMA). Standards contained 1–100 mM l-Arginine, 0.5–5 mM ADMA, and 0.5–5 mM SDMA. 

### 2.7. Renal H_2_S-Releasing Activity

The H_2_S-releasing activity of the kidney was measured using the methylene blue method as described earlier [12]. Concentration was calculated against a calibration curve of NaHS (3.125–250 µM) and represented as μM/gram protein/min. All samples were run in duplicate.

### 2.8. Immunohistochemistry Staining for 8-OHdG

8-Hydroxydeoxyguanosine (8-OHdG) is a DNA oxidation product that was determined to detect DNA damage. As we described previously [14], paraffin-embedded tissue sectioned at 4 μm thickness was deparaffinized in xylene and rehydrated in a graded ethanol series to phosphate-buffered saline. Following blocking with immunoblock (BIOTnA Biotech., Kaohsiung, Taiwan), the sections were incubated for 2 h at room temperature with an anti-8-OHdG antibody (1:100, JaICA, Shizuoka, Japan). Immunohistochemical staining was detected using the polymer-horseradish peroxidase (HRP) labelling kit (BIOTnA Biotech) and 3,3′-diaminobenzidine (DAB) as the chromogen. A negative control of identical staining omitting incubation with a primary antibody was used. Quantitative analysis of 8-OHdG-positive cells per microscopic field in the renal sections was performed as we described previously [14].

### 2.9. Statistical Analysis

All data are presented as mean ± the standard error of the mean. Statistical analyses were performed using one-way ANOVA or two-way ANOVA where appropriate. Tukey’s post hoc test was applied where multiple comparisons were made. BP was analyzed by two-way repeated-measures ANOVA and Tukey’s post hoc test. Bioinformatics analyses of gut microbiota were performed using R software. Based on the normalized OTU abundance profile, microbial α-diversity was measured by one-way ANOVA followed by false discovery rate (FDR) correction using the ACE index. The dissimilarity of the microbial communities among groups was evaluated by PLS-DA using R software. Sample clustering in β-diversity analysis was tested by ANOSIM using the vegan package in R software. The key bacterial taxa responsible for discrimination between different groups were identified using the linear discriminant analysis effect size (LEfSe) algorithm. The linear discriminant analysis (LDA) score threshold > 3 and *p* < 0.05 indicated significantly enriched microbial communities. The significance level was set at 5% level. Statistical analyses were performed using SPSS software (SPSS Inc., Chicago, IL, USA). 

## 3. Results

### 3.1. Body Weight and Blood Pressure of Male Offspring 

Table 2 shows there was no mortality in any group. The body weight (BW) and kidney weight (KW) of the LC group were lower compared to the control, while the KW-to-BW ratio was comparable among the four groups. 

**Figure 1:**
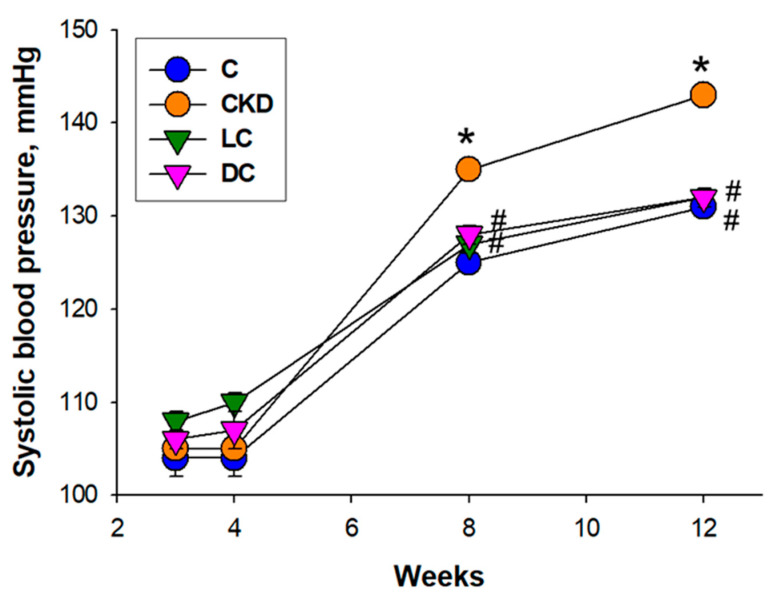
Effect of maternal chronic kidney disease (CKD), l-cysteine (LC), and d-cysteine (DC) on systolic blood pressures in male rat progeny from 3 to 12 weeks of age. *n* = 8/group; * *p* < 0.05 vs. C; # *p* < 0.05 vs. CKD.

The BP of rat offspring measured between week 8 and week 12 showed that maternal CKD caused a higher systolic BP (SBP) vs. controls, in which it was prevented by l- or d-cysteine supplementation (Figure 1). At 12 weeks old, SBP, diastolic BP, and mean arterial pressure (MAP) were higher in the CKD group than those in the controls. Taken together, observations from Figure 1 and Table 2 demonstrated that CKD caused hypertension in adult offspring, which l- or d-cysteine supplementation prevented. 

### 3.2. H_2_S Signaling Pathway

**Figure 2:**
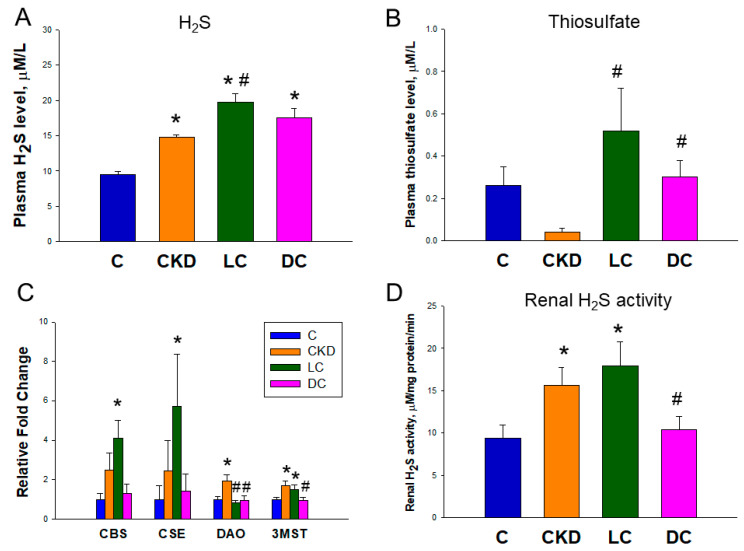
Effect of maternal chronic kidney disease (CKD), l-cysteine (LC), and d-cysteine (DC) on (**A**) plasma H_2_S level, (**B**) plasma thiosulfate level, (**C**) renal mRNA expression of H_2_S-generating enzymes, and (**D**) renal H_2_S-releasing activity. *n* = 8/group; * *p* < 0.05 vs. C; # *p* < 0.05 vs. CKD.

Results for the H_2_S signaling pathway were shown in Figure 2. The CKD group showed an increase in plasma H_2_S level that was significantly enhanced by l-cysteine treatment in the LC group (Figure 2A). Compared to CKD group, a higher plasma thiosulfate level was observed in the LC and DC groups (Figure 2B). 

Renal transcript abundance of H_2_S-generating enzyme CBS, CSE, DAO, and 3MST was compared in Figure 2C. Compared to the control, transcript levels of CBS and CSE were higher in the LC group. Maternal CKD increases transcript abundance of DAO and 3MST. These increases were reduced by d-cysteine treatment. Renal H_2_S-releasing activity is compared in Figure 2D. Maternal CKD significantly increased renal H_2_S-releasing activity in the CKD and LC, while d-cysteine treatment reduced it. To summarize, these findings suggest that maternal CKD caused a compensatory increase of certain H_2_S-generating enzyme expression and activity in the offspring’s kidneys, thereby increasing the plasma concentration of H_2_S. 

l-cysteine therapy enhanced H_2_S and thiosulfate production and was related to the increase of renal mRNA expression of CBS and CSE and H_2_S-releasing activity. d-cysteine protected offspring against maternal CKD-induced hypertension and coincided with increased thiosulfate. Thiosulfate is an intermediate in oxidative H_2_S metabolism, which can alternatively be reduced and regenerate H_2_S [19]. Increased thiosulfate not only serves as a means of increased recycling H_2_S but also conserves biologically relevant thiols to counterbalance oxidative stress. 

### 3.3. Tryptophan Metabolites

**Figure 3:**
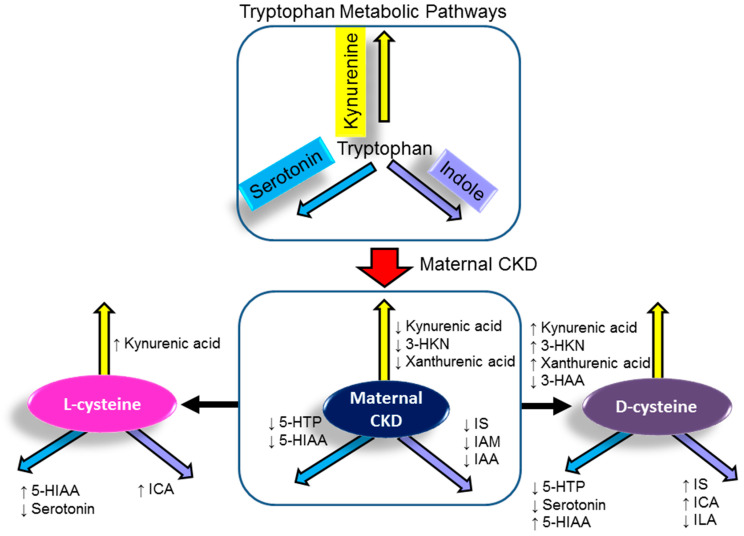
Effect of maternal chronic kidney disease (CKD), l-cysteine, and d-cysteine on major tryptophan-derived metabolites. ↑ = increase; ↓ = decrease. 3-HKN = 3-hydroxykynurenine; 3-HAA = 3-hydroxyanthranilic acid; 5-HTP = 5-hydroxytryptophan; 5-HIAA = 5-hydroxyindole acetic acid; IS = Indoxyl sulfate; IAM = Indole-3-acetamide; ILA = indolelactic acid; ICA = Indole-3-carboxaldehyde; IAA = indoleacetic acid.

We determined tryptophan and its metabolites in the plasma using LC-MS in three major metabolic pathways: the kynurenine pathway, the serotonin pathway, and the indole pathway. In 12-week-old offspring, no difference was found in the plasma level of tryptophan between the four groups (Table 3). Nevertheless, maternal CKD significantly reduced plasma levels of kynurenic acid, 3-HKN, xanthurenic acid, 5-HTP, 5-HIAA, IS, IAM, and IAA. The decreases of kynurenic acid and 5-HIAA were restored by l- or d-cysteine supplementation. Additionally, d-cysteine supplementation increased plasma levels of 3-HKN, xanthurenic acid, but decreased 3-HAA compared to those in the CKD group. In serotonin metabolic pathways, both l- and d-cysteine similarly reduced 5-HTP and serotonin, while they increased 5-HIAA levels. Moreover, l-cysteine caused lower plasma IS, IAM, and IAA levels than controls in the indole metabolic pathway. There were higher plasma levels of IS and ICA in the DC group compared to the controls. A schematic summarizing how maternal CKD, l-cysteine, and d-cysteine supplementation altered the major tryptophan metabolites is presented in Figure 3.

### 3.4. Oxidative Stress

**Figure 4:**
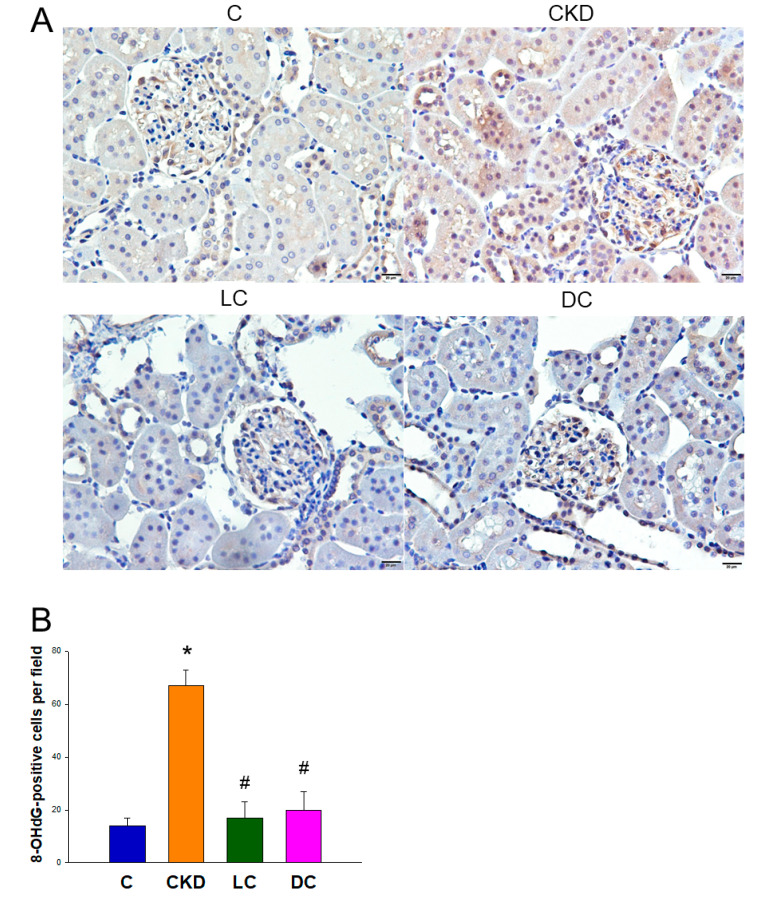
(**A**) Light micrographs illustrating immunostaining for 8-hydroxydeoxyguanosine (8-OHdG) in the offspring kidneys exposed to maternal chronic kidney disease (CKD), l-cysteine (LC), or d-cysteine (DC). (**B**) Quantitative analysis of 8-OHdG-positive cells per microscopic field (×200). *n* = 8/group; * *p* < 0.05 vs. C; # *p* < 0.05 vs. CKD.

We next evaluated 8-OHdG staining in the kidney by using immunohistochemistry. In the glomeruli and renal tubules, 8-OHdG showed intense staining in the CKD group (67 ± 6 positive cells), while little staining in the LC group (17 ± 6 positive cells), DC group (20 ± 7 positive cells), and controls (14 ± 3 positive cells) (Figure 4).

### 3.5. Alterations in Microbiome

**Figure 5:**
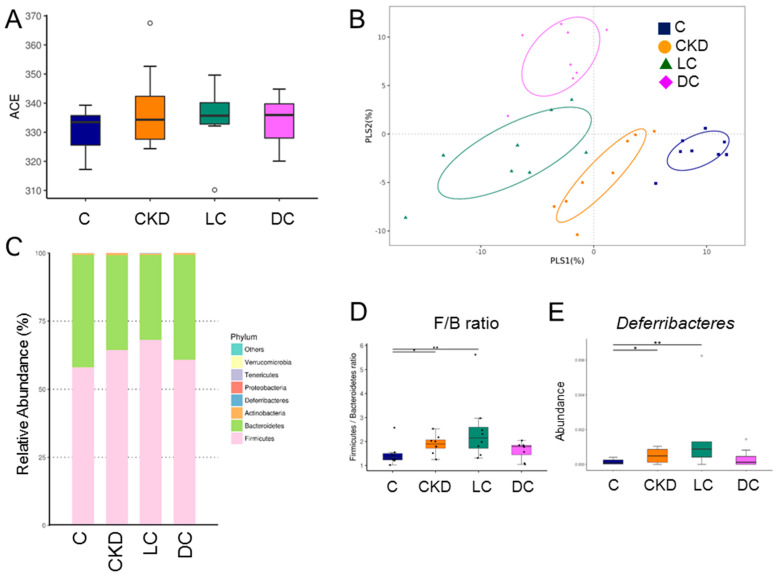
Effect of maternal chronic kidney disease (CKD), l-cysteine (LC), and d-cysteine (DC) on the gut microbiome. (**A**) α-diversity measured by abundance-based coverage estimator (ACE) index. (**B**) β-diversity using the Partial Least Squares discriminant analysis (PLS-DA). (**C**) Relative abundance of the top five phyla of the gut microbiota. (**D**) The *Firmicutes* to *Bacteroidetes* (F/B) ratio. (**E**) Relative abundance of the phylum *Deferribacteres*. Data are shown as means ± SEM; *n* = 8/group. * *p* < 0.05; ** *p* < 0.01.

Results for gut microbiota composition are shown in Figure 4. Microbial α-diversity (ACE index) did not differ between the four groups (Figure 5A). We next compared the microbial community similarity using two β-diversity measures, the PLS-DA and ANOSIM. Scatterplots of PLS-DA analysis are depicted in Figure 5B and show significant clustering according to study group, indicating that the microbial community was distinctly altered by different interventions. The ANOSIM analysis also confirmed a significant difference between the four groups (All *p* < 0.05). Figure 5C illustrated the major bacterial phyla present in offspring’s microbiomes, including *Firmicutes, Bacteroidetes, Actinobacteria*, *Deferribacteres*, and *Proteobacteria*. Compared to the controls, the *Firmicutes*/*Bacteroidetes* (F/B) ratio, a microbial marker associated with hypertension [20], was higher in the CKD and LC groups (Figure 5D). Similarly, the phylum *Deferribacteres* proportion was greater in the CKD and LC groups compared with the controls (Figure 5E). 

**Figure 6:**
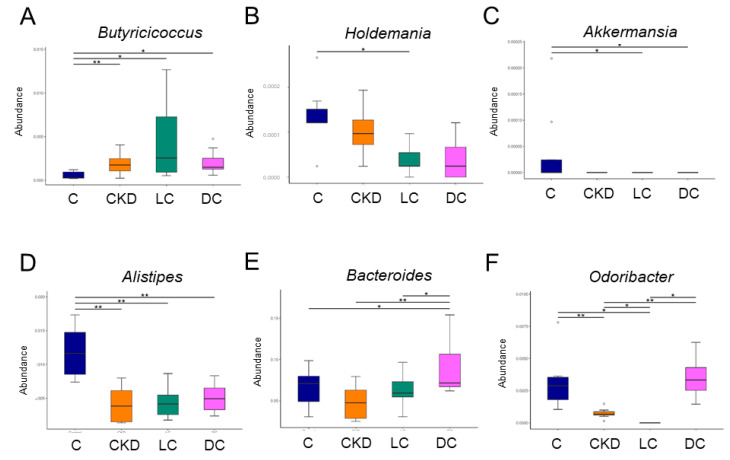
Effect of maternal chronic kidney disease (CKD), l-cysteine (LC), and d-cysteine (DC) on the gut microbiome at the genus level. Relative abundance of the genera (**A**) *Butyricicoccus*, (**B**) *Holdemania*, (**C**) *Akkermansia*, (**D**) *Alistipes*, (**E**) *Bacteroides*, and (**F**) *Odoribacter*. *n* = 8/group. * *p* < 0.05; ** *p* < 0.01.

Compared to the C group, genus *Butyricicoccus* abundance was higher in the other three groups (Figure 6A). l-cysteine therapy reduced the proportion of the genus *Holdemania* in the LC group compared with the controls (Figure 6B). As a result, relative abundance of genus *Akkermansia* was significantly lessened by l- or d-cysteine therapy (Figure 6C). In addition, the proportion of *Alistipes* was greater in the C group compared to the other three groups (Figure 6D). Moreover, d-cysteine caused an increase in the abundance of genus *Bacteroides* in the DC group compared with the other three groups (Figure 6E). d-cysteine treatment restored the reduction of genus *Odoribacter* abundance caused by CKD (Figure 6F). Together, these results indicated that the protective effects of d-cysteine were associated with increased abundance of genera *Bacteroides* and *Odoribacter*, while the indole-producing genera *Alistipes* and *Akkermansia* were relatively depleted in response to d- or l-cysteine treatment.

**Figure 7:**
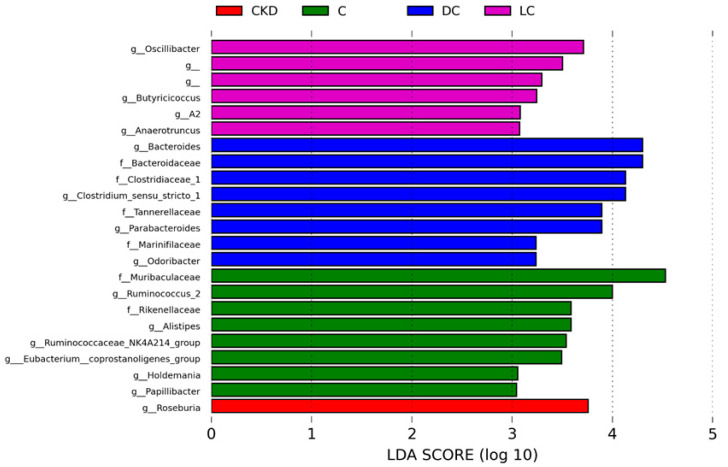
Linear discriminant analysis effect size (LEfSe) was assessed for biomarker discovery. Most enriched bacterial taxa in the C (green), CKD (red), LC (purple), and DC (blue) groups are illustrated. The linear discriminant analysis (LDA) score threshold was set to greater than 3.

Results for the LEfSe algorithm are depicted in Figure 7. The LEfSe analysis indicated a greater proportion of genus *Roseburia* in the CKD group. Certain taxa, like genera *Oscillibacter* and *Butyricicoccus*, were significantly enriched in the l-cysteine-treated CKD offspring. d-cysteine treatment resulted in an enriched genera proportion of *Bacteroides* and *Odoribacter*. 

### 3.6. NO Pathway

Plasma NO parameters are compared in Table 4. Compared to the controls, plasma l-Arginine level and the l-Arginine-to-ADMA ratio were lower in the CKD group. The reduction was improved by the l-cysteine supplementation. Additionally, ADMA and SDMA levels did not differ between the four groups. These results therefore indicate that, in CKD rats, NO pathway was impaired and characterized as a decreased l-Arginine and the l-Arginine-to-ADMA ratio. Conversely, decreased NO bioavailability was improved by l-cysteine therapy.

### 3.7. Renin–Angiotensin System

**Figure 8:**
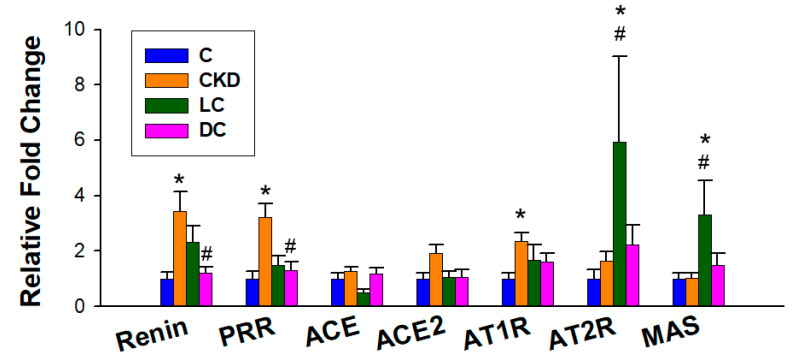
Effect of maternal chronic kidney disease (CKD), l-cysteine (LC), and d-cysteine (DC) on the renin–angiotensin system. *n* = 8/group. * *p* < 0.05 vs. C; # *p* < 0.05 vs. CKD.

We further evaluated the RAS genes by qPCR (Figure 8). CKD increased renal mRNA expression of the renin and (pro)renin receptor (PRR), which was restored by d-cysteine treatment. Renal AT1R expression was induced by CKD, which was partially prevented by l- or d-cysteine treatment. Additionally, the l-cysteine treatment significantly induced increases of renal AT2R and MAS expression. 

## 4. Discussion

Our study affords new insights into the beneficial effects of maternal l- or d-cysteine therapy to protect against maternal CKD-induced offspring hypertension with specific emphasis on H_2_S signaling pathways and tryptophan metabolites derived from gut microbes. Our main findings are described as follows: (1) maternal CKD-induced hypertension was similarly prevented by l- or d-cysteine supplementation in gestation; (2) l-cysteine therapy protected adult offspring against hypertension and was related to an increase in plasma H_2_S and thiosulfate levels; (3) compared to CKD, d-cysteine treatment increased tryptophan metabolites in the kynurenine pathway, but decreased those in the serotonin pathway; (4) the protective effect of both l- and d-cysteine was associated with the reduction of renal oxidative stress, represented as 8-OHdG staining; (5) maternal CKD and l- and d-cysteine treatments differentially shaped offspring’s gut microbiota profiles, resulting in four distinct enterotypes; (6) the beneficial effects of d-cysteine were relevant to the increase of genera *Bacteroides* and *Odoribacter* abundance; (7) the beneficial effect of l-cysteine was associated with the restoration of l-Arginine levels and the l-Arginine-to-ADMA ratio in the plasma; and (8) both l- and d-cysteine therapy protected offspring hypertension programmed by maternal CKD coinciding with rebalancing the RAS. 

In support of our prior study in SHRs [14], l- and d-cysteine supplementation revealed similar BP-lowering effects in adult offspring born to mothers with CKD. Of note, l- or d-cysteine supplementation was administered to mother rats during pregnancy, therefore the reduction of BP in adult offspring was due to reprogramming, instead of an acute effect. Our study provides further evidence that early-life supplementation with specific amino acids could reverse the programming processes and provide benefits regarding hypertension [21].

The reduction of BP observed in this study is consistent with previous findings demonstrating the vasorelaxant properties of H_2_S [10,11,22]. H_2_S can be endogenously produced using substrate l- or d-cysteine [10,11,22]. From our data, l-cysteine treatment increased renal H_2_S-generating enzyme CBS and CSE expression, renal H_2_S-releasing activity, as well as plasma levels of H_2_S and thiosulfate. d-cysteine restored CKD-induced reduction of plasma thiosulfate levels, while it had little effect on renal H_2_S-generating enzymes. Conflicting with a previous study reporting that the renal d-cysteine pathway is 80-fold greater at H_2_S-producing activity than the l-cysteine pathway [23], our results revealed that they both involve differential regulation of the H_2_S-generating pathway but their beneficial effects are comparable.

The benefits of l- and d-cysteine may involve their ability to modulate the gut microbiome, including enhancing the abundance of certain beneficial microbes and the mediation of tryptophan-metabolizing bacteria. A higher α-diversity has been shown more beneficial for hypertension [19]; however, we observed that α-diversity did not differ among the four groups. Although an increased F/B ratio observed in the CKD group is consistent with previous findings displaying this ratio could serve as a microbial marker associated with hypertension [19], our data also showed an increased ratio in the LC group without hypertension. 

The data in this work revealed that l- or d-cysteine supplementation enhanced the abundance of several beneficial bacteria like *Butyricicoccus, Bacteroides*, and *Odoribacter* spp. [24,25]. This result was unsurprising in view of a previous study showing the abundance of the butyrate-producing genus *Odoribacter* was inversely correlated with BP [26]. The beneficial effects of l- or d-cysteine on hypertension reprogramming, at least in part, are associated with the enhancement of beneficial microbes.

Our data demonstrated that maternal CKD and cysteine treatment had differential effects on offspring’s tryptophan metabolites derived from the indole and serotonin pathways. Notably, our data showed that maternal CKD caused a reduction of plasma IS, IAM, and IAA levels, all of which are indole derivatives. Both IS and IAA are well-known uremic toxins derived from tryptophan, which can bind the aryl hydrocarbon receptor (AHR) whose activation is related to an increased risk of hypertension [27]. Activation of AHR signaling can trigger oxidative stress and inflammation [6,7,28,29,30], by which tryptophan-derived uremic toxins are closely associated with the development of cardiovascular disease. Exposure to AHR ligands has been shown to enhance the expression of ROS-generating enzymes, increase ROS production, trigger pro-inflammatory T helper 17 axis, and induce pro-inflammatory cytokines production [29,30]. Therefore, additional studies are required to clarify whether the interplay between tryptophan-derived uremic toxins and AHR plays a role in the pathogenesis of programmed hypertension via induction of oxidative stress and inflammation. The decreases of IS, IAM, and IAA observed in offspring born to CKD dams coincided with hypertension, suggesting that decreases of indole metabolites might be an offsetting mechanism but not a cause of CKD-induced hypertension.

Several types of intestinal bacteria have been implicated in tryptophan metabolism [31,32,33], such as *Alistipes, Akkermansia*, and *Bacteroides*. We found the indole-producing genera *Alistipes* and *Akkermansia* were relatively depleted in response to l-cysteine treatment. As l-cysteine reduced *Alistipes* and *Akkermansia* at the genus level, the decreases of tryptophan metabolites IS, IAM, and IAA were probably due to the decreased abundance of indole-producing gut microbes. Considering H_2_S can regulate microbial tryptophanase activity to affect the degradation of tryptophan to indole [11,16], our results demonstrate the feasibility of altering the production of indole metabolites through manipulation of the gut microbiota by l-cysteine treatment. Furthermore, we observed that both l- and d-cysteine similarly reduced plasma serotonin levels. One previous study reported that formula-diet-driven microbiota could shift the tryptophan metabolic pathway from serotonin to tryptamine, which coincided with increased genus *Butrycimonas* but decreased *Holdemania* and *Akkermansia* [34]. Therefore, additional studies are needed to elucidate how H_2_S mediates certain tryptophan-metabolizing microbes to direct different metabolic pathways of tryptophan. 

Prior research has shown that the beneficial effects of H_2_S on hypertension might be due to a resetting of new balance between vasoconstrictors (e.g., RAS) and vasodilators (e.g., NO) [12,13]. Our data in this work demonstrated that l-cysteine not only improved NO bioavailability but also increased AT2R and MAS. It is known that AT2R and MAS are part of the protective arm of the RAS, which can counterbalance the deleterious effects mediated by Angiotensin II (Ang II) [35]. On the other hand, d-cysteine reduced renal mRNA expression of renin, PRR, and AT1R. Considering the renin/PRR axis and Ang II/AT1R axis both promote hypertension, it is possible that d-cysteine could influence the RAS towards its BP-lowering benefit. 

Another protective mechanism of l- and d-cysteine therapy on programmed hypertension in this model may be associated with the reduction of oxidative stress. We observed that both l- and d-cysteine therapy improved CKD-induced oxidative stress in the offspring’s kidneys, represented as 8-OHdG staining. Our data is consistent with prior research demonstrating that oxidative stress is involved in the pathogenesis of programmed hypertension during kidney development [8]. 

Some limitations of this study should be acknowledged. Firstly, we mainly focused on the kidneys. Hence, very little was known about what role other BP-controlled organs play in the beneficial effect of l- or d-cysteine against maternal CKD-induced hypertension. Secondly, we did not examine microbiota changes at various stages of development. Gut microbial changes in adult progeny may reflect postnatal plasticity instead of a primary programmed process in responding to maternal CKD and cysteine supplementation. Furthermore, to our knowledge, no studies have been reported for simultaneous determination of all tryptophan metabolites. Although our developed method can quantify 13 metabolites belonging to three different tryptophan metabolic pathways, there are still some important metabolites, such as quinolinic acid and melatonin, which are excluded. Additional studies are required to improve the method for monitoring most tryptophan metabolites and how the metabolism of tryptophan varies between the three metabolic pathways, which could provide insight into CKD and related diseases. Lastly, considering the complex tryptophan metabolism in microbiota-host crosstalk, determining which tryptophan metabolites mainly promote the beneficial effect of maternal l- and d-cysteine treatment deserves further investigation. 

## 5. Conclusions

In conclusion, the results of the present study indicate that dietary supplementation with l- or d-cysteine protects adult offspring against maternal CKD-induced hypertension. These beneficial effects of cysteine supplementation were associated with the enhancement of H_2_S production, enrichment of beneficial microbes, alterations of tryptophan-metabolizing bacteria and tryptophan metabolites, reduction of oxidative stress, restoration of NO bioavailability, and rebalancing of the RAS. Tryptophan metabolites may act as mediators of the gut–kidney communication, and there is an urgent need for studies on the regulation of tryptophan metabolism via altering gut microbiota in CKD. Moving toward a greater understanding of the mechanisms behind H_2_S and tryptophan metabolism implicated in the programming of hypertension is critical to developing ideal reprogramming intervention to halt the global epidemic of hypertension.

## Figures and Tables

**Table 1:** Primer sequences for quantitative real-time PCR.

Gene	5′ Primer	3′ Primer
*CBS*	5 atgctgcagaaaggcttcat 3	5 gtggaaaccagtcggtgtct 3
*CSE*	5 cgcacaaattgtccacaaac 3	5 gctctgtccttctcaggcac 3
*3MST*	5 ggctcagtaaacatcccattc 3	5 tgtccttcacagggtcttcc 3
*DAO*	5 ccctttctggaaaagcacag 3	5 ctcctctcaccacctcttcg 3
*Renin*	5 aacattaccagggcaactttcact 3	5 acccccttcatggtgatctg 3
*PRR*	5 gaggcagtgaccctcaacat 3	5 ccctcctcacacaacaaggt 3
*ACE*	5 caccggcaaggtctgctt 3	5 cttggcatagtttcgtgaggaa 3
*ACE2*	5 acccttcttacatcagccctactg 3	5 tgtccaaaacctaccccacatat 3
*AT1R*	5 gctgggcaacgagtttgtct 3	5 cagtccttcagctggatcttca 3
*AT2R*	5 caatctggctgtggctgactt 3	5 tgcacatcacaggtccaaaga 3
*MAS*	5catctctcctctcggctttgtg 3	5 cctcatccggaagcaaagg 3
*R18S*	5 gccgcggtaattccagctcca 3	5 cccgcccgctcccaagatc 3

**Table 2:** Weight and blood pressure of male offspring.

Groups	C	CKD	LC	DC
Mortality	0%	0%	0%	0%
Body weight (BW) (g)	384 ± 7	367 ± 12	305 ± 9 *,#	363 ± 6
Left kidney weight (g)	1.67 ± 0.05	1.62 ± 0.08	1.36 ± 0.06 *,#	1.70 ± 0.06
Left kidney weight/100 g BW	0.43 ± 0.01	0.44 ± 0.01	0.44 ± 0.01	0.47 ± 0.01
Systolic BP (mmHg)	131 ± 1	143 ± 1 *	132 ± 1 #	132 ± 1 #
Diastolic BP (mmHg)	91 ± 1	98 ± 2 *	84 ± 2 #	90 ± 2 #
Mean arterial pressure (mmHg)	104 ± 1	113 ± 2 *	100 ± 1 #	104 ± 2 #

*n* = 8/group; BP= blood pressure. * *p* < 0.05 vs. C; # *p* < 0.05 vs. CKD.

**Table 3:** Plasma levels of tryptophan metabolites.

Groups	C	CKD	LC	DC
Tryptophan (ng/mL)	22,856 ± 946	22,400 ± 1216	21,499 ± 611	20,778 ± 807
Kynurenic acid (ng/mL)	7.53 ± 0.61	5.46 ± 0.52 *	8.64 ± 1.15 #	7.2 ± 0.33 #
3-HKN (ng/mL)	6.77 ± 0.29	5.18 ± 0.22 *	5.38 ± 0.19 *	7.06 ± 0.31 #
Xanthurenic acid (ng/mL)	3.71 ± 0.32	2.71 ± 0.21 *	4.04 ± 0.67	3.52 ± 0.26 #
3-HAA	4.02 ± 0.4	3.01 ± 0.39	4.13 ± 0.41	1.45 ± 0.27 *,#
5-HTP (ng/mL)	6.42 ± 0.21	5.61 ± 0.23 *	5.29 ± 0.28 *	4.49 ± 0.17 *,#
Serotonin (ng/mL)	329 ± 121	120 ± 18	26 ± 5 *#	21 ± 10 *,#
5-HIAA (ng/mL)	16.3 ± 0.8	13.1 ± 0.7 *	17.2 ± 0.8 #	16.2 ± 1 #
N-AS (ng/mL)	3.92 ± 0.79	2.76 ± 0.32	2.52 ± 0.51	3.3 ± 0.72
IS (ng/mL)	3066 ± 184	1974 ± 153 *	2312 ± 189 *	3285 ± 420 #
IAM (ng/mL)	84.6 ± 4.1	69.4 ± 3 *	70.3 ± 3.6 *	81.6 ± 6.3
ILA (ng/mL)	282 ± 8	272 ± 13	262 ± 6	238 ± 6 *,#
ICA (ng/mL)	2.73 ± 0.39	2.18 ± 0.24	4.26 ± 0.72 #	5.45 ± 1.04 *,#
IAA (ng/mL)	84.6 ± 4.1	69.4 ± 3 *	70.3 ± 3.6 *	81.6 ± 6.3

*n* = 8/group; * *p* < 0.05 vs. C; # *p* < 0.05 vs. CKD. 3-HKN = 3-hydroxykynurenine; 3-HAA = 3-hydroxyanthranilic acid; 5-HTP = 5-hydroxytryptophan; 5-HIAA = 5-hydroxyindole acetic acid; N-AS = N-acetylserotonin; IS = Indoxyl sulfate; IAM = Indole-3-acetamide; ILA = indolelactic acid; ICA = Indole-3-carboxaldehyde; IAA = indoleacetic acid.

**Table 4:** Plasma NO parameters.

Groups	C	CKD	LC	DC
l-Arginine (μM)	355.3 ± 11.9	267.2 ± 8.5 *	330.6 ± 14.3 #	287.4 ± 37.5
Asymmetric dimethylarginine (μM)	2.15 ± 0.08	2.17 ± 0.13	1.89 ± 0.23	2.06 ± 0.25
Symmetric dimethylarginine (μM)	2.15 ± 0.11	2.39 ± 0.18	2.12 ± 0.14	1.92 ± 0.08
l-Arginine-to-ADMA ratio (μM/μM)	167.3 ± 8.7	125.5 ± 6.1 *	197.8 ± 30.4 #	156.6 ± 29.6

*n* = 8/group; * *p* < 0.05 vs. C; # *p* < 0.05 vs. CKD.

## Data Availability

Data is contained within the article.
